# One Biosecurity: a unified concept to integrate human, animal, plant, and environmental health

**DOI:** 10.1042/ETLS20200067

**Published:** 2020-10-28

**Authors:** Philip E. Hulme

**Affiliations:** The Bio-Protection Research Centre, Lincoln University, PO Box 85084, Canterbury, New Zealand

**Keywords:** biological invasions, biosecurity, epidemiology, One Health

## Abstract

In the wake of the SARS-CoV-2 pandemic, the world has woken up to the importance of biosecurity and the need to manage international borders. Yet strong sectorial identities exist within biosecurity that are associated with specific international standards, individual economic interests, specific research communities, and unique stakeholder involvement. Despite considerable research addressing human, animal, plant, and environmental health, the science connections between these sectors remain quite limited. One Biosecurity aims to address these limitations at global, national, and local scales. It is an interdisciplinary approach to biosecurity policy and research that builds on the interconnections between human, animal, plant, and environmental health to effectively prevent and mitigate the impacts of invasive alien species. It provides an integrated perspective to address the many biosecurity risks that transcend the traditional boundaries of health, agriculture, and the environment. Individual invasive alien plant and animal species often have multiple impacts across sectors: as hosts of zoonotic parasites, vectors of pathogens, pests of agriculture or forestry, as well as threats to biodiversity and ecosystem function. It is time these risks were addressed in a systematic way. One Biosecurity is essential to address several major sociological and environmental challenges to biosecurity: climate change, increasing urbanisation, agricultural intensification, human global mobility, loss of technical capability as well as public resistance to pesticides and vaccines. One Biosecurity will require the bringing together of taxonomists, population biologists, modellers, economists, chemists, engineers, and social scientists to engage in a new agenda that is shaped by politics, legislation, and public perceptions.

## One Biosecurity: time to progress invasion science beyond One Health

**Figure 1.**
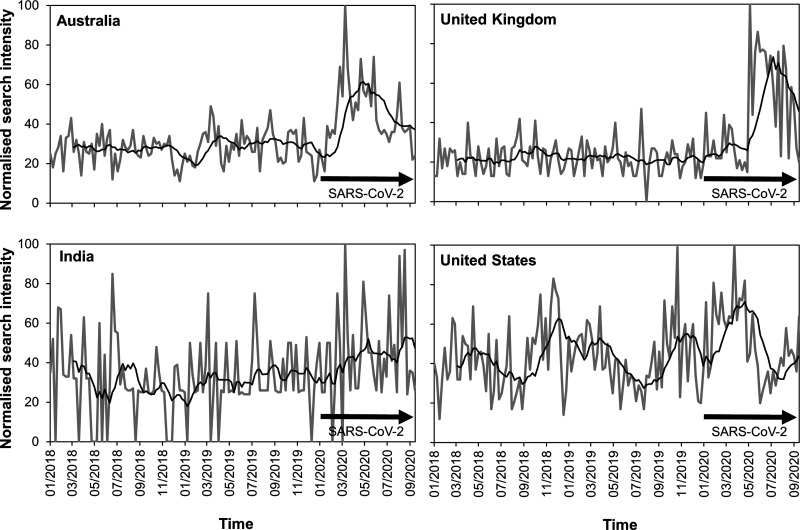
Temporal trend in the interest over time in biosecurity since January 2018 as captured by a sample of web searches submitted to Google® and captured by Google Trends data for Australia, India, the U.K. and the United States. Data are provided for actual search intensity each week (grey lines) and a 3 month rolling average (black lines). In each case, a clear increase in global interest in biosecurity coincided with the SARS-CoV-2 pandemic in early 2020. Data represent the results of a search for the term ‘biosecurity’ on 21/08/20 and have been normalised to scale between 0 and 100.

In the wake of the SARS-CoV-2 pandemic, much of the world has become aware of the importance of biosecurity in protecting global health (Figure 1) and in particular the need for more effective management of the risks of introducing unwanted organisms at international borders [1,2]. This has led several countries to review their national biosecurity systems in order to gain from the lessons arising from the pandemic [3–5]. Yet the lessons for implementing more robust biosecurity strategies are much broader than most policymakers currently appreciate, going far beyond the actions undertaken in response to SARS-CoV-2. Although biosecurity is sometimes viewed as a synonym of biodefense or biosafety [6], it more commonly refers to the research, procedures, and policies that cover the exclusion, eradication, or effective management of the risks posed by the introduction of alien plant pests, animal pests and diseases, animal diseases capable of transmission to humans (zoonoses), the release of genetically modified organisms and their products, and the management of invasive alien species and genotypes [7,8]. Although this definition is often adopted in multilateral policy guidance [9–11], at an international level the issues encompassed by biosecurity have traditionally been dealt with by different sectors each with its own regulatory framework. The World Health Organisation (WHO), the International Plant Protection Convention (IPPC), and the World Organisation for Animal Health (OIE) provide international standards for human health, plant health, and animal health, respectively, while the Convention on Biological Diversity (CBD) sets non-binding standards for the management of alien species that threaten biodiversity [8,12,13]. These divisions are often echoed at the national level, with most governments developing domestic regulation relating to human health, agriculture and the environment through separate departments, ministries, or agencies [14,15].

These sectorial divisions appear not to recognise the fundamental similarities in the processes underpinning biological invasions whether they relate to plant, animal, or human health and this may, in part, reflect the lack of cross-disciplinary thinking among the research community [16,17]. Recent assessments have pointed out similar dramatic increases in the rate at which plants, animals and microorganisms have been transported around the world over the last 100 years [18]. Although SARS-CoV-2 has prompted a re-appraisal of human diseases as biological invasions [2], this underplays the fact that some of the earliest invasive alien species were undoubtedly the parasites and pathogens of humans [19]. The causative agent of leprosy (*Mycobacterium leprae*) appears to have originated in Eastern Africa or the Near East over 2000 years ago and subsequently spread with successive human migrations to Europe and Asia, then with European colonists who introduced leprosy into West Africa and the Americas in the 18th century and the Pacific in the 19th century [20]. Viral diseases such as smallpox, measles, mumps, and polio have undergone a similar trajectory, moving across the globe within a few centuries in the wake of international trade [21]. Furthermore, the global distribution of many zoonotic livestock diseases that are infectious to humans, such as highly pathogenic avian influenza, Q fever, and anthrax, have been facilitated by human trade in poultry, cattle, pigs, and horses [22]. Thus, the global redistribution of pathogens, parasites, plants, and animals should all be viewed as biological invasions [19] and since they share many fundamental aspects of their spatial and temporal dynamics [16,23,24] a more integrated approach to biosecurity is warranted.

To be effective, biosecurity policies must take advantage of a more coherent, universal approach that seeks synergies between the health, agriculture, and environment sectors at national and international levels and should aim to shift the traditional focus on regulating individual organisms and sectors to ensuring confidence in the overall risk management framework [25]. Progress towards a more holistic approach to biosecurity has been recognised by several recent initiatives. The One Health movement was initiated in 2007 with the aim of bringing veterinary and human health closer together since the divide between veterinarians and doctors was seen as an obstacle to addressing the many new or re-emerging human diseases that come from animals [26]. Although One Health encompasses environmental health, the definition is fairly narrow in that the environment is viewed primarily in terms of how it can influence the rate of disease emergence and transmission to humans such as via tropical deforestation and/or climate change [27]. In 2014, the Global Health Security Agenda (GHSA) was launched, committing over 20 governments to strengthen the capacities of national and local organisations to prevent, detect and respond to infectious disease threats [28]. Although the GHSA aims to operationalise the concept of One Health, it has a strong focus on improved vaccination programmes and reducing antimicrobial resistance with less of an emphasis of the environment and socioeconomic drivers of human health [29]. More recently, the Planetary Health Alliance (PHA) has sought to determine the human health consequences of anthropogenic disruptions of Earth's natural systems [30,31]. The PHA includes a much broader view of environmental health that includes the need to ensure food security and secure access to safe drinking water, yet this huge breadth of coverage and the complexity of the systems encompassed in environmental health could certainly limit the implementation of the PHA [32]. Nevertheless, the One Health, GHSA, or PHA initiatives do not explicitly address the core issues of biosecurity, specifically the global proliferation of alien pests, weeds, and pathogens that are inextricably intertwined and are significant threats to human wellbeing as well as public and environmental health. Here, the concept of One Biosecurity is proposed and elaborated for the first time in the scientific literature in order to present the strong synergies among plant, animal, human, and environmental health and the urgent need for a more integrated treatment of biosecurity threats. One Biosecurity is an interdisciplinary approach to biosecurity policy and research that builds on the interconnections between human, animal, plant, and environmental health to prevent and mitigate the impacts of invasive alien species more effectively. However, biosecurity is an incredibly broad topic that encompasses threats to food security from plant pests, loss of endangered species due to alien predators, the spread of transgenes from genetically modified crops as well as the risks of biological contaminants in food [7]. Thus in order to focus on the key principles, and given the considerable global concern regarding emerging diseases and future pandemics [33,34], this review illustrates the value of the One Biosecurity approach with particular reference to human health.

## One Biosecurity captures the synergies in human, animal, plant, and environmental health

Currently, strong sectorial identities exist within biosecurity that are associated with specific international standards, individual economic sectors such as health, agriculture and the environment, specific research communities and unique stakeholder involvement. For example, human biosecurity addresses zoonotic and emerging disease diagnosis and investigation; animal biosecurity deals with disease prevention and control in livestock production holdings, aquaculture farms, and feed storage facilities; plant biosecurity aims to safeguard plant industries and crop production; whereas environmental biosecurity is concerned with the protection of the environment and social amenity from the negative effects associated with invasive alien species [8]. Such divisions work well when addressing the problems of biological invasions that are exclusive to a sector such as specialist pathogens, parasites, or pests that affect only a single host species. However, the links between human, animal, plant, and environmental biosecurity have been little explored but are can often be substantial [7].

**Figure 2.**
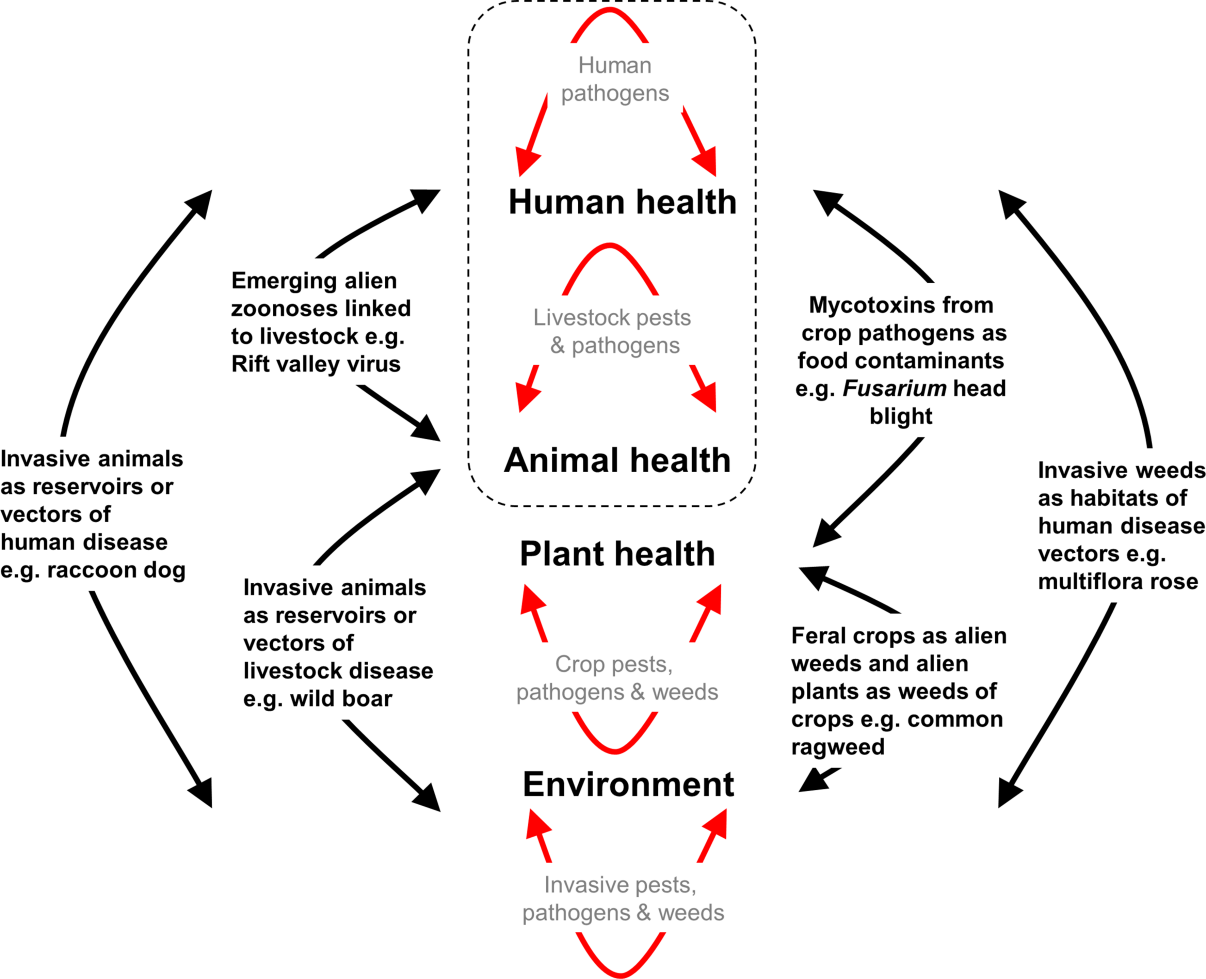
Schematic representation of the One Biosecurity concept emphasising the links between human, animal, plant, and environmental health arising through the impacts of invasive alien plants, animals, and pathogens. The dashed rectangle represents the sectors that are the focus of One Health and highlights the more comprehensive aspect of One Biosecurity.

One Biosecurity provides a unified framework to address the many biosecurity risks that transcend the traditional boundaries of animal health, plant health, human health, and the environment (Figure 2). There are many examples where an alien species has impacts across multiple sectors including the environment and human health. The raccoon dog (*Nyctereutes procyonoides*) originates in the Far East but is now a widespread invasive canid species in Northern, Eastern, and Central Europe, where it not only competes successfully with native predatory mammals but is also a vector of wildlife, livestock, and human diseases [35]. Similarly, the wild boar (*Sus scrofa*) has been introduced to the Americas and Oceania where it destroys crops, is associated with declines in threatened and endangered species, and contributes to the transmission of a wide variety of parasites, viruses, and bacteria that can infect humans and domestic livestock [36]. Cross-sectorial impacts are not limited to vertebrates, invertebrates can also have impacts across human and livestock health. The giant African snail (*Achatina fulica*) has been introduced widely throughout the tropics where its feeding leads to considerable crop losses which are exacerbated by its role as a vector of plant pathogens (*Phytophthora* spp.), it outcompetes native gastropods and is also an intermediate host playing a role in the transmission of *Angiostrongulus* spp. the causative agents of eosinophilic meningoencephalitis in livestock and humans [37]. Invasive alien vectors of disease can also have wider impacts. The Asian tiger mosquito (*Aedes albopictus*) is able to transmit at least 22 arboviruses to wild and domesticated animals as well as humans and thus can impact the population viability of wildlife as well as facilitate the spread of zoonotic diseases, such as Rift Valley virus from livestock to people [38]. However, while the role that invasive alien plants, invertebrates and vertebrates play in human health has become increasingly recognised [39–41], with the exception of alien mosquitoes, current understanding of the role of alien species in the transmission of zoonotic diseases between livestock and humans remains limited.

In forest ecosystems, invasive alien plants not only impact upon biodiversity and forestry activities but can also directly impact human and livestock health by facilitating the transmission of pathogens and parasites. The understory structure provided by invasive alien shrubs such as Japanese barberry (*Berberis thunbergii*) and multiflora rose (*Rosa multiflora*) provide a buffered microclimate that limits desiccation-induced mortality of the blacklegged tick (*Ixodes scapularis*) increasing their density as well as the prevalence of infection with the spirochete *Borrelia burgdorferi* the causative agent of Lyme disease that affects both human and livestock health [42,43]. High rates of pathogen transmission will occur where the main reservoir hosts also preferentially associate with the increased cover provided by invasive alien shrubs. Not all cross- sectoral impacts need be through the spread of disease. Due to its painful sting, the red imported fire ant (*Solenopsis invicta*) causes significant impacts on human and livestock health, but it also directly destroys crops at the seedling stage and dramatically reduces the diversity of native invertebrates through predation [44]. A high alien weed density in small grain cereal crops can reduce yields but also increase the prevalence of the alien fungal disease fusarium head blight that leads to shrunken, low quality grains as well as results in the contamination of grains with mycotoxins that are hazardous to animal and human health [45]. Common ragweed (*Ambrosia artemisiifolia*) reduces crop yield, outcompetes native vegetation and produces large quantities of allergenic pollen that is a significant cause of asthma and rhinitis in humans [46].

**Figure 3.**
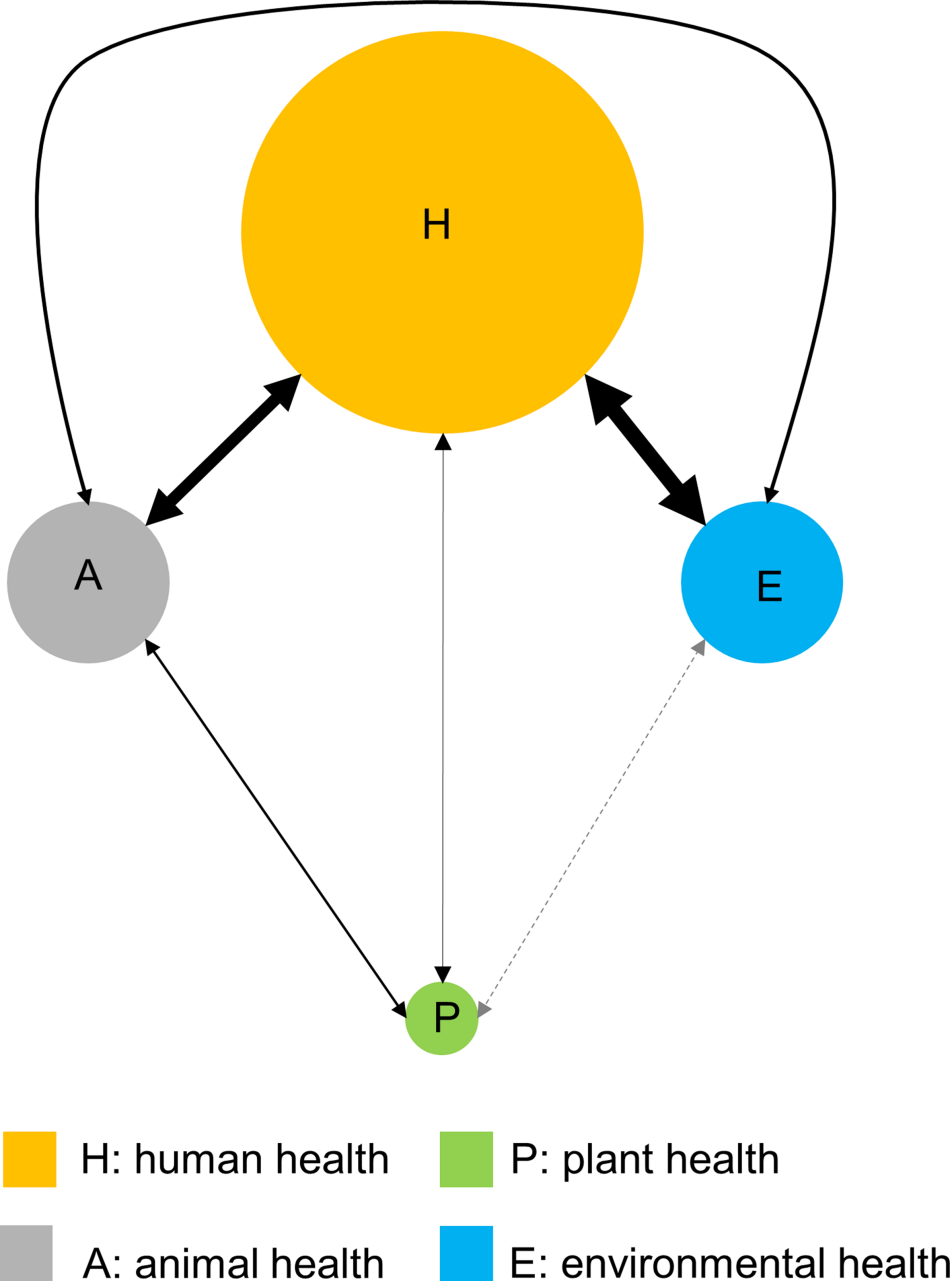
Network diagram describing the links captured by the scientific literature addressing the topics of human health (H), animal health (A), plant health (P), and environmental health (E). The size of the circles is proportional to the number of papers published on that topic while the size of the arrows is proportional to the number of papers that address the two adjoining topics. Data are from a literature search using Web of Science over the period 1980–2019 undertaken on 21/08/20 using the individual search terms ‘human health’, ‘animal health’, ‘plant health,’ and ‘environmental health’ either on their own or in pairs.

The foregoing examples highlight that a key aim of One Biosecurity is to drive a more holistic research agenda that examines the impacts of invasive alien species across the health, agriculture, and the environment sectors. Understandably, not all species will necessarily have impacts across all these sectors, but many will undoubtedly impact at least two. However, despite considerable research addressing human, animal, plant, and environmental health, the science connections between these sectors remain quite limited and much less than would be expected given the volume of outputs for each individual sector (Figure 3). The strongest connections are between the sectors traditionally associated with One Health, but even here less than 1% of published research in the fields of human and animal health address connections between these sectors.

## One Biosecurity addresses future challenges for health, agriculture, and the environment

The world is facing major sociological, political, and environmental predicaments that require robust strategies and responses. To illustrate why a One Biosecurity approach is needed, five major challenges are examined in order to highlight the need for coordinated action: urbanisation, human mobility, capability deficits, agricultural intensification, and management constraints.

Urban areas with high human population density appear to have been hardest hit by the SARS- CoV-2 pandemic [47]. It is expected that the urbanisation of rural regions and the increasing growth of megacities is likely to lead to a heightened risk of pest and disease incursions as well as the emergence of new zoonoses due to the increasing interaction of urban communities with invasive alien species. Human dominated ecosystems, such as urban regions, host a greater diversity of zoonotic host species [48]. As a consequence, urban development is one of the main drivers of disease spillover from wildlife to humans and domestic animals, but also from domestic animals to wildlife [49]. Furthermore, alien species comprise an increasingly significant proportion of biodiversity in urban ecosystems [50]. Many alien bird and mammal species in urban environments have zoonotic potential [51] and this indicates a potential for high rates of transmission either directly to humans or via domesticated animals. Yet for most vertebrates introduced around the world, their effectiveness as reservoir hosts of zoonotic pathogens is unknown. This represents a significant gap in the management of future zoonotic risk.

The projected increases in the international movement of people and goods, particularly from areas that present higher biosecurity risks, will further complicate the ability to identify biosecurity risks at international borders. In 2013, there were over one billion tourists moving between different countries around the world and this has been forecast to reach 1.8 billion by 2030 with tourists now increasingly visiting regions away from traditional holiday destinations [52,53]. This will result in the potential for tourists to not only inadvertently introduce alien species into new environments but also become exposed to zoonotic agents. Airline baggage is an important route through which alien insect pests can enter new regions [54], while soil on footwear and sports equipment can introduce plant pathogens [52]. In Europe, the Asian tiger mosquito has been responsible for outbreaks of dengue in France, chikungunya virus in Italy and West Nile Virus in Greece as a result of a single infected human host returning from overseas and subsequently facilitating disease transmission [55]. The importance of international travel in the spread of invasive alien species has been underscored in the effectiveness of strict travel and border control measures on the global spread of SARS-CoV-2 [56]. However, such drastic action is not sustainable in the long-term thus integrated solutions to manage all sources of risk arising from international travellers must be developed in the future.

The SARS-CoV-2 pandemic has highlighted the lack of preparedness of many countries to deal with a health crisis on such a large scale and the shortage of appropriately trained public health officials [57]. A critical issue facing all areas of biosecurity is the skill shortages in key areas such as taxonomy, microbiology, and entomology that place significant limitations on the ability to develop biosecurity systems and respond to pest and disease incursions. The development of formal, internationally recognised, qualifications that deliver high standards of professional excellence in biosecurity is viewed as an essential step to respond to the increasing exposure of society and the environment to biological threats [58]. Although in many areas professional qualifications already exist [59,60], they are rarely sufficiently comprehensive to meet contemporary biosecurity challenges. Academic institutions and employers must recognise that biosecurity is a multidisciplinary field that draws on a wide range of subjects including epidemiology, pathobiology, economics, social behaviour, and invasion science. The SARS-CoV- 2 pandemic has revealed that biosecurity professionals need not only to have an understanding of risk management in containment facilities, but also virus pathogenesis, the role of non-human hosts in the emergence of zoonotic diseases, the implications of global travel and trade on disease spread as well as the social challenges of disease mitigation and control. Rather than relying on a set of narrowly focused credentials, implementing a much broader multidisciplinary curriculum as a foundation for biosecurity professionals will be essential to strengthen the world's ability to prevent, detect, and respond to invasive alien species threats worldwide.

The increasing intensification of agriculture to supply an ever greater demand from the world's population for more food [61] will affect the ability to contain and limit the spread of a pest or disease following an incursion or outbreak. The role that the intensification of livestock production could play in future animal and human disease pandemics is well recognised by national and international bodies [62]. While agricultural intensification is known to be a potential trigger for pest outbreaks in agricultural crops [63,64], the strategies to mitigate such risks are not well developed. There is, therefore, considerable opportunity for cross-fertilization in best practice across plant and animal health. More importantly, it is generally underappreciated how disease risks to human health can also impact global food security. Epizootic pandemics such as avian flu or African swine fever directly reduced animal-sourced food output, while restrictions in the international movement of agricultural labourers in response to SARS-CoV-2 have disrupted supply chains and increased the volatility of commodity prices on the international market [65]. The strong interrelationships between human, animal, plant, and environmental health associated with food production require joined up thinking across the health, agriculture, and environment sectors when building greater global food security.

Despite the high profile of health risks arising from the SARS-CoV-2 pandemic there remains substantial concerns regarding the likely levels of compliance (even among medical workers) should an effective vaccine become available [66]. Similarly, the effective management of pest animals, weeds, and agricultural pathogens is becoming increasingly difficult. At least two global trends will challenge existing approaches to the management of invasive alien species, including human and livestock diseases. The first trend is the increasing resistance of invasive alien plants, invertebrates, vertebrates, and pathogens to methods of chemical control, whether in the form of pesticides, fungicides, herbicides, or antimicrobials [67–71]. Strategies to combat resistance to chemical control strategies are vital to ensure the sustainability of current invasive alien species management, and this requires research to find alternative mode-of-action synthetic chemical pesticides and other nonchemical approaches. However, the rate at which new pesticides and antimicrobials are being registered is slower than the rate at which active ingredients are being removed from the market and may limit the ability to control pests, weeds, and pathogens [72,73]. The second trend is the social resistance to the tools used to combat pests, weeds, and pathogens. The management of vertebrate pests depends on the use of traps, pesticides, repellents, and other methods, each of which can cause varying levels of pain and other negative experiences to animals. Increasingly the humaneness of control techniques is viewed as more important than its effectiveness resulting in humane but less efficient tools being available for pest control [74]. The public are increasingly resistant to pesticides in the environment and in their food, putting increasing pressure on pest control to apply more environmentally acceptable alternatives and/or reduce rates of application [75]. At the same time, there remains a growing social movement of public health vaccine opposition [76,77]. In the United States, an increasing number of antivaccine activities are being established in major metropolitan areas, rendering select cities vulnerable for vaccination-preventable diseases [77]. This is a particularly alarming development since urban areas are likely to be hotspots for the emergence of new zoonotic diseases. The consequences of these two global trends is that post-border biosecurity management will become increasingly complex, challenging scientists to develop new tools to replace currently unacceptable approaches and compelling society to appreciate the cost and benefits of pest and disease control. Given the parallels in these trends facing both human and animal health as well as plant and environmental health, a unified approach under the banner of One Biosecurity would certainly bring benefits.

These five challenges illustrate the need for the greater integration promoted by the One Biosecurity concept. Many other similar challenges exist that require a unified approach, not least the impact of climate change which will not only have a direct effect on the biology of pathogens, plants, invertebrate, and vertebrates and their constituent ecosystems [49,78,79] but also will frame the human responses that might exacerbate these direct effects. Furthermore, climate change will interact with several of the challenges listed above such as urbanisation, human mobility, and agricultural intensification. These trends emphasise the urgency of adopting a One Biosecurity perspective.

## One Biosecurity: a better way forward

The earliest conceptualisation of One Biosecurity stems from a review of Australia's quarantine and biosecurity arrangements undertaken in 2008 that sought to encourage a stronger partnership between the federal and state governments underpinned by a legal framework to support national responses to alien pests and diseases relevant to agriculture [15]. However, as has been shown in the previous sections, One Biosecurity must build on the interconnections among the health, agriculture, and environment sectors, have relevance at global, national and local scales and be interdisciplinary by embracing the natural and social sciences. By defining One Biosecurity in these broader and more enlightened terms the potential for transformative change in the management of invasive alien species is much more likely. A One Biosecurity perspective will require the bringing together of taxonomists, population biologists, modellers, economists, chemists, engineers, and social scientists to engage in an agenda that is shaped by politics, legislation, and public perceptions. However, One Biosecurity could easily suffer the same criticisms faced by One Health that it is no more than a buzzword and that in most cases the disciplinary divisions remain [80]. How might this be avoided?

The first step is to ensure that the development of One Biosecurity sits front and centre of international and national policies. Under the SPS Agreement, World Trade Organization Members have the right to adopt sanitary and phytosanitary measures necessary for the protection of human, animal, and plant life or health [81]. These measures must be science-based, not more trade restrictive than required and not arbitrarily or unjustifiably discriminatory against trading partners. This clearly argues for a holistic approach that embraces the health, agriculture, and environment sectors as captured by One Biosecurity. While this should mean that assessments need to capture the risks to human, animal, and plant life, at present the cross-sectorial nature of biological invasions is not captured effectively in the tools used for assessing biosecurity risks. For example, national and international animal health panels only examine the risks of introducing species that might directly or indirectly harm animal health [82], while risk assessments of plant pests (weeds, insects, and pathogens) focus on impacts on crop yields and ecosystem services [83] and those for invasive alien species examine impacts on biodiversity [84]. Building interdisciplinary risk assessment tools should be a priority but is not without its challenges. It is likely that the environmental and social costs of biological invasions will be dwarfed by those of the human health and agriculture sectors, even where costs of control and eradication are similar and this will make the weighing up of risks more complex. For example, in New Zealand, estimates of the total expected costs of all established alien plants on the environment over 10 years amounted to only one-third of the likely annual cost of managing a foot-and-mouth disease outbreak [7]. However, the development of these tools will also be an effective means of bringing different disciplines together, allowing the comparison of different risk assessment protocols to derive an optimum hybrid approach that might be a radical departure from current techniques and catalyse interdisciplinary research agendas to fill the many knowledge gaps.

Developing a common risk assessment approach while essential, will not in itself drive the adoption of One Biosecurity. This first requires common understanding among scientists and policymakers of the shared threats invasive alien species pose to animal, plant, human, and environmental health. An initial mechanism may be to raise awareness of the benefits of One Biosecurity through the Intergovernmental Science-Policy Platform on Biodiversity and Ecosystem Services (IPBES) which has recently launched a specific assessment on the impacts of invasive alien species, including threats to human health and quality of life [85]. With sufficient momentum, a specific International Convention addressing One Biosecurity could provide the essential governance oversight across existing multilateral agreements and conventions to ensure a more co-ordinated and synergistic approach to global biosecurity. However, such an option would need to overcome increasing resistance to multilateral initiatives [86] which could be possible if the wider economic benefits as well as cost-effectiveness of an International Biosecurity Convention are made self-evident. However, the concept of One Health has gained momentum even in the absence of a dedicated International Convention [87]. In the absence of a multilateral support, nation states such as New Zealand and Australia that already have a strong biosecurity regulations could lead the way in developing national One Biosecurity frameworks which, if successful, could catalyse other nations to follow suit. Time will tell how feasible these options might be but hopefully it will not take another global pandemic for the logic of One Biosecurity to be realised.

## Summary

- Biosecurity is increasingly important in an ever more connected world that is exposed to multiple threats that impact human health, agriculture, and the environment sectors, yet policy is strongly sector specific.
- One Biosecurity bridges these sectors to allow greater foresight in the management of invasive alien plants, animals and pathogens that impact human health, animal health, plant health, and the environment.
- Many invasive species impact multiple sectors but to date their overall threat to society and the economy are insufficiently well captured by current risk assessment tools resulting in unforeseen outcomes.
- The major future challenges to biosecurity such as urbanisation, climate change, agricultural intensification, and increased human mobility all require the more holistic understanding provided by One Biosecurity to achieve a greater cross-fertilization of ideas and an improvement in approaches to deal with threats that impact multiple sectors.

## Abbreviations

CBD: Convention on Biological Diversity
GHSA: Global Health Security Agenda
IPBES: Intergovernmental Science-Policy Platform on Biodiversity and Ecosystem Services
IPPC: International Plant Protection Convention
PHA: Planetary Health Alliance
WHO: World Health Organisation
OIE: World Organisation for Animal Health
